# Correspondence

**Published:** 1912-03

**Authors:** S. A. Grove, F. W. Busche, Chas. H. Derr, J. S. Greey

**Affiliations:** Chairman


### CORRESPONDENCE

*To the Medical Profession*:

Dear Doctor:

You are probably familiar with the action of the Bell Telephone Co. in arbitrarily reducing commissions for booth space and service from 40 per cent. to 10 per cent. cancelling our contracts and removing telephone booths, because we would not meekly toe the mark.

The Bell Co. gives less and receives more than the Frontier Co. all along the line, for the Bell charges a guarantee of $65.00 per year, the Frontier only $48.00

The Bell Co. charges 5 cents for each outgoing message, the Frontier installs desk phone with free service, Bell Co. is trying to force us to sign a 10 per cent. contract for both space and service, while the Frontier gives 40 per cent. and says it is well worth it.

Now as professional men who are in close touch with us, we ask your co-operation and advice in this fight, for we realize that you are a power both as to personal influence and also in regards numbers. So if as individuals or through your associations, you are able to bring some pressure to bear on the Bell Telephone Co. your friendly offices would be surely appreciated by all Pharmacists in New York State for New York City and Rochester are going through a similar experience and are doing all in their power to resist the unjust demands of this telephone corporation.

We remain, yours truly, Special Telephone Committee,

S. A. Grove, Chairman,
F. W. Busche,
Chas. H. Derr,
J. S. Greey,

